# A Hybrid Deep Learning-Driven SDN Enabled Mechanism for Secure Communication in Internet of Things (IoT)

**DOI:** 10.3390/s21144884

**Published:** 2021-07-16

**Authors:** Danish Javeed, Tianhan Gao, Muhammad Taimoor Khan, Ijaz Ahmad

**Affiliations:** 1 Software College, Northeastern University, Shenyang 110169, China; 2027016@stu.neu.edu.cn; 2 Riphah Institute of Science and Engineering, Islamabad 44000, Pakistan; mtaimoor.khan@infosecurity.com.pk; 3 Institute of Computer Sciences and Information Technology (ICS/IT), The University of Agriculture, Peshawar 25130, Pakistan; ijaz@siat.ac.cn

**Keywords:** Internet of Things (IoT), intrusion detection, deep learning (DL), software-defined network (SDN)

## Abstract

The Internet of Things (IoT) has emerged as a new technological world connecting billions of devices. Despite providing several benefits, the heterogeneous nature and the extensive connectivity of the devices make it a target of different cyberattacks that result in data breach and financial loss. There is a severe need to secure the IoT environment from such attacks. In this paper, an SDN-enabled deep-learning-driven framework is proposed for threats detection in an IoT environment. The state-of-the-art Cuda-deep neural network, gated recurrent unit (Cu- DNNGRU), and Cuda-bidirectional long short-term memory (Cu-BLSTM) classifiers are adopted for effective threat detection. We have performed 10 folds cross-validation to show the unbiasedness of results. The up-to-date publicly available CICIDS2018 data set is introduced to train our hybrid model. The achieved accuracy of the proposed scheme is 99.87%, with a recall of 99.96%. Furthermore, we compare the proposed hybrid model with Cuda-Gated Recurrent Unit, Long short term memory (Cu-GRULSTM) and Cuda-Deep Neural Network, Long short term memory (Cu- DNNLSTM), as well as with existing benchmark classifiers. Our proposed mechanism achieves impressive results in terms of accuracy, F1-score, precision, speed efficiency, and other evaluation metrics.

## 1. Introduction

In recent years, there has been an enormous growth in the Internet of Things (IoT), described as a global network of interconnected devices that are assigned unique addresses. IoT devices use different communication protocols and sensing features. These devices have computational abilities to analyze data and provide services. IoT is an archetype connecting millions of digital intelligent devices, prompting the formation of an intelligent atmosphere i.e., smart factories, smart ecosystems, intelligent health systems, smart cities, and vehicular networks [1]. However, besides leveraging huge benefits, IoT also presents various security concerns and evolving threats. Due to the rapid growth of data in IoTs, a considerable number of attacks and threats are also focused on IoT networks [2,3]. IoT contains heterogeneous and homogeneous networks with networking devices that use different types of protocols. It means that vulnerabilities can produce an imperceptible threat to IoT devices and the entire system. Cybersecurity exploits numerous concerns in the dynamic features of these devices in the form of different attacks, i.e., DoS attacks, DDoS attacks, and some other types of malware [4]. In a single day, about 80% of cybersecurity experts try to handle at least one security issue, while 60% of experts deal with the network’s operations and security for an hour or two per day [5]. Deception attacks and replay attacks have also been described. Industrial level security controls and attack detection techniques are reviewed in [6]. There are various kinds of protocol-following devices, and different security mechanisms need to be implemented for each device. However, In the seamless nature of IoT devices, these security measures are insufficient. To secure the complete IoT infrastructure, there hasn’t been invented an integrated approach yet. IoT security remains a significant challenge and poses a severe need for security.

Nowadays, SDN-enabled framework not only enhances the abilities of dynamic and heterogeneous environment of IoT but also deliver the opportunity to simplify the network management. It provides efficient and effective detection without exhaustion and provides a platform for underlying resource-constrained devices that do not overburden a security solution. For SDN surveillance, one of the best approaches is integrating IDS in SDN [7]. With the rapid evolution of AI along with the programmable features of SDN, security levels can be improved by integrating SDN into AI-based security solutions. Many techniques based on AI have been employed as network traffic algorithms that have shown certain levels of accuracy and ideal results, i.e., genetic algorithms, *k*-nearest neighbor, ANNs, decision trees, naive Bayesian, and fuzzy logic [8]. To sum up, the need to present a robust and flexible architecture for threat detection in IoT devices encourage us to propose an SDN-enabled, deep-learning-based intrusion detection solution.

### 1.1. Contribution

The main contributions of the paper are as follow:

- SDN-enabled deep-learning-driven solution is proposed that is highly cost-effective and scalable for threats detection in IoT environment.
- Cu-DNNGRU + Cu-BLSTM classifiers are used for effective threat detection in IoTs.
- Cu-GRULSTM and Cuda- Cu-DNNLSTM are exploited on the same data set to compare ur results.
- For verification purpose, the proposed mechanism is compared with the existing literature works for a better performance evaluation under CICIDS data set.
- Finally, 10 folds cross-validation is conducted in this research to show the unbiasedness of our results.
- The evaluation results show that the proposed mechanism is able to provide a multiclass detection, and outperforms in terms of detection accuracy and computational complexity.

### 1.2. Organization

The remaining paper is designed as follows. Section 2 comprises related work and background. In Section 3, the proposed methodology, data set description, and other details are elaborated. Section 4 presents the evaluation metrics and experimental setup. The results is discussed in detail in Section 5. Finally, we conclude the paper in Section 6.

## 2. Related Work

SDN is emerging as a capable next-generation network framework. It comprises three layers known as data, control, and application plane with their APIs (i.e., northbound and southbound). The SDN’s control plane has the capability of extending many networks in the SDN’s data plane, e.g., internet of things, fog, edge [9,10]. The control plane can adapt to different functionalities, and it is completely programmable. It deals with the heterogeneity of IoT nodes among SDN controllers and linked devices of IoT through Open-Flow switches. In SDN’s design, the control and data plane are separated, allowing flexibility and simplification. Furthermore, it provides the central control functions and network’s global view, simplifying the collection of network statistics [11]. Thus SDN provides dynamism, scalability, and centralized management. It plays an essential role in improving control decisions. It is recognized as a chief and flexible enabler for network solutions [12]. Integration of SDN and IoT provides an accurate approach for inspecting networks to identify threats, malware, suspicious activities, and attacks. Therefore, SDN pledges a promising future for the (IoT).

Researchers have proposed different techniques and threat detection schemes in the existing literature. In [13], the authors presented an IDS for a network that uses a convolutional neural network (CNN). The authors in [14] proposed a group of recurrent families for attacks and threat detection in IoT by analyzing network traffic using long shortterm memory (LSTM) on Modbus-TCP network traffic data. For attack identification and classification, a recurrent neural network (RNN) is used in [15]. Furthermore, a comparison is made by the authors by comparing non RNN techniques with RNN. The authors in [16] used Random Forests (RF) classifiers trained on a self-generated data set using Wireshark for the DDoS attacks detection in IoT. Support vector machine (SVM) classifier is trained on a data set provided by Defense Advanced Research Projects Agency (DARPA) for IDS in SDN’s [17]. In [18], the authors aim to identify the compromised intelligent devices in an IoT ecosystem by presenting a self-learning system. They used a Gated recurrent unit (GRU) classifier for the detection of compromised devices. The authors in [19] used LSTM for botnet detection using Czech Technical University’s real-time traffic (CVUT). In [20], the authors used Bayesian, J48, and Naïve Bayes to detect Internet Relay Chat (IRC) botnet. However, the authors didn’t mention anything about their detection accuracy in their work. The authors in [21] used LSTM for categorizing attacks from regular traffic. Multilayer ANN anomalies detection in a network is presented in [22]. The authors claim that their proposed work is capable of detecting DoS attacks with an accuracy of 99.4%. In [23], the authors used a deep model for the detection of distributed attacks in the IoT network. They achieved an accuracy of 98.27% by training the algorithm on the NSL-KDD data set. A deep-learning-driven SDN-based framework is used in [24] for securing IoT infrastructure. The authors used the KDD99 data set to train Restricted Boltzmann Machine (RBM) with a detection accuracy of 95%. In [25], the authors proposed a flow-based detection mechanism in the gateway of SDN for the mitigation and detection of DoS attacks. However, this work lacks efficiency analysis and proven performance.

Artificial-intelligence-based techniques are beneficial in recent years by integrating them with SDN for threat detection [26]. The authors in [27] proposed an intrusion detection system with training and testing accuracy of 96.22% and 92.73%. The model first ranks the security aspects by defining their relevancy and then establishes IDS based on the maximum related ones. In [28], the authors used SVM, DNN, NaiveBayes, and j48 classifiers for intrusion detection. These classifiers are trained on the NSL-KDD data set. They proposed that DNN is better in comparison to other classifiers. The authors in [29] proposed a framework for botnet investigation at packet level detection in IoT. The authors used CNN and RNN classifier, trained on CTU-13 and ISOT data sets, and achieved an accuracy of 99.3%. In [30], the authors proposed SDN-based, bio-inspired IDS for crossfire attacks with a detection accuracy of 80%. The authors in [31] used a DL-driven method called DeepDefence to detect DDoS traffic. A Number of DL models are used to classify benign traffic and attacks traffic. In addition, the authors used the Blocked-Recurrent-Unit-Neural-Network (GRU), the LSTM, CNN, and RNN and accomplished a decent cut in the rate of conventional approaches. In [32], the authors used DL and SDN to tackle DDoS attacks, and the results showed 99% and 98% accuracy with the ISCX data set. The authors in [33] presented a source-based defense mechanism on DDoS on the hogzilla data set and achieved up to 98.88% accuracy. In [34], the authors introduces a DDoS attack detection system based on multilevel deep learning technology. The whole system, the intelligent network, aims for more accurate and efficient detection of DDoS attacks. In [35], authors adopted a progressive transfer learning approach for DDoS problems and achieved improved performance than the current methods. The authors in [36] presented the DADMCNN framework through in-depth learning to detect DDoS attacks. In addition, the authors proposed an MC-CNN model to maximize feature information for better recognition. The authors in [37] proposed an automatic learning approach based on SDN capabilities. Advanced learning methods using ANN, LSTM, and CNN to build the learning model. In addition, the performance of the proposed model will be assessed using the Mininet Wi-Fi emulation platform. Authors in [38] used LSTM to construct a deep neural network model and add an Attention Mechanism for enhancement of performance and achieved 96.2% of accuracy. The authors in [39] presented a combined framework by using real network data and CNNs for early detection of DDoS by performing experiments on open CDR data set released by Italia Telecom consisting of over 319 million CDRs. Results indicate that the projected framework achieved more than 91% detection of underattack cells and normal accuracy. A novel CNN architecture based on categorical crossentropy is presented by emerging a multilayer convolution feature-fusion mechanism along with a loss on the NSLKDD data set in [40]. Experimental results demonstrate that the proposed model offer improved accuracy with low-false-alarm. However, network structure requires optimization to attain better detection results. In [41], the authors proposed a CNN-based anomaly detection technique for DDoS attacks using the CAIDA data set. Authors presented anomaly detection technique achieved 87.35% accuracy in detection of DDoS attack. DL-based codetection model along with Snort IDS is presented in [42] for detection of IoT-based DDoS attacks. Authors performed experiments on a data set collected from network-based traffic by different tools and achieved 95% of detection accuracy of TPR along with less than 4% of FPR. In [43], the authors presented a new realistic BoT–IoT data set. The data set was developed on a realistic testbed, and it contains simulated and legitimate IoT network traffic with different types of attacks. The authors in [44] presented a data set named as MQTTset, which is related to the MQTT protocol. The authors implemented different machine learning algorithms to validate the data set. Furthermore, they compared the results of the balanced and unbalanced data set. Upon comparison, the unbalanced data set reported a high accuracy due to a high number of records for benign. Finally, a labeled behavioral data set of IoT is generated in [45], which incorporates benign and malicious traffic. The data set is generated from real-time traffic in a medium-sized network, i.e., a network of 83 devices. The existing literature is presented in detail in Table 1.

## 3. Methodology

This research work aims to propose a hybrid DL-driven framework for intrusion detection in IoT devices. This part of the paper describes the proposed work methodology, i.e., proposed DL-driven hybrid framework, proposed network model, data set description, and preprocessing.

### 3.1. Proposed Network Model

**Figure 1:**
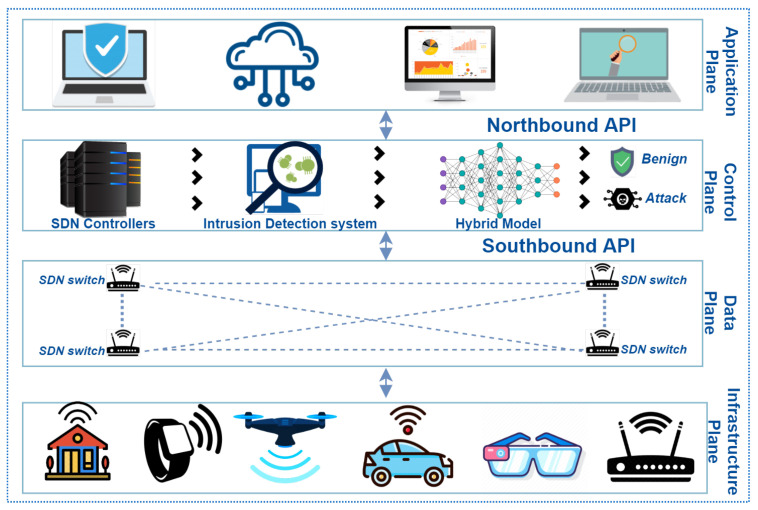
Proposed Network Model.

In recent years, SDN came up as integrated network design technology. In SDN’s design, the control plane and data plane are separated, allowing flexibility and simplification. Furthermore, it provides the central control functions and network’s global view, which simplifies the collection of network statistics. We propose hybrid DL-driven, SDN-enabled architecture for intrusion and threat detection in the environment of IoT. The proposed hybrid model (Cu-DNNGRU + Cu-BLSTM) is placed in the control plane, as shown in Figure 1. There are multiple reasons for placing the hybrid threat detection model in the control plane: Firstly, this plane of SDN is entirely programmable as well as SDN has the capability of extending IoT devices on its data plane. Secondly, it uses open-flow switches, which provide solutions for heterogeneity between SDN controllers and IoT devices. Thirdly, the control plane can leverage the primary devices of IoT without the exhaustion that makes it a proper revolution for IoT. The integration of SDN and IoT proposes a suitable way to inspect network traffic to identify attacks, threats, and unauthorized events. The proposed framework is centralized and highly cost-effective. Furthermore, the data plane of SDN consists of numerous IoT devices, i.e., smart devices, sensors, and other wireless technologies.

### 3.2. Hybrid DL-Driven Detection Scheme

**Figure 2:**
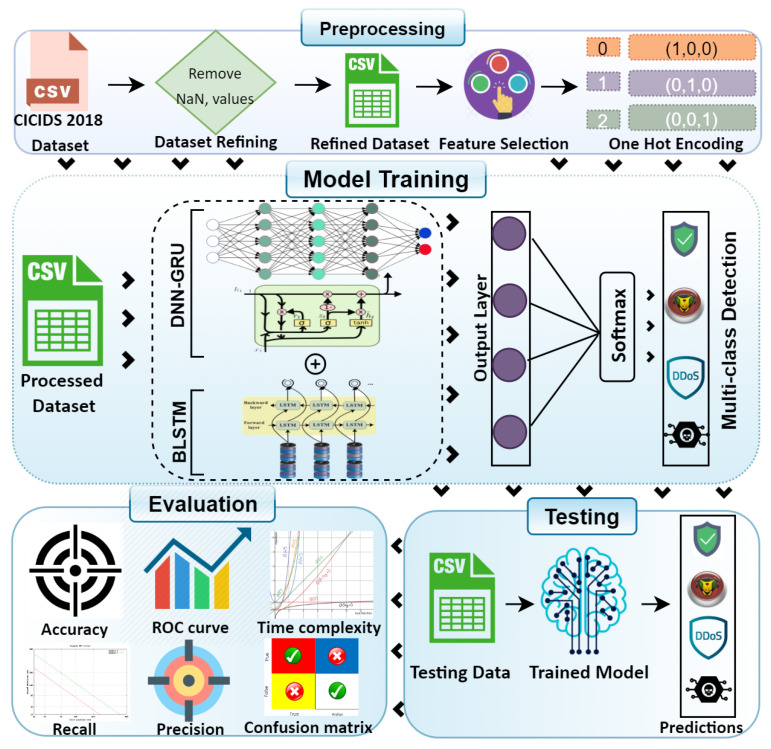
Proposed hybrid detection framework.

The authors offer a DL-driven hybrid framework for intrusion detection in IoT. The DL-driven Cu-DNNGRU + Cu-BLSTM is used for threat detection in IoT networks. A cost-effective, versatile, and powerful threat detection module is developed to detect multiclass threats. Figure 2 depicts a complete overview of the proposed model. The proposed scheme consists of CU-DNNGRU and Cu-BLSTM models for intrusion detection and detects sophisticated threats and malware in IoT environments. The proposed model is tested and trained on hybrid algorithms with low false positives (FP) and greater detection accuracy. The model consists of different layers, i.e., Cu-DNNGRU comprises one layer with 200 neurons. However, Cu-BLSTM has one layer with 100 neurons. We have used softmax as an activation function in the output layer, and in other layers, the Relu function is used. For achieving efficient results, we have performed the experimentation till five epochs with batch sizes of 32. For experimentation, we have used Cuda-enabled versions with the processing of GPU for improved performance.

Furthermore, the proposed work utilized Keras framework with the backend of Tensor Flow for Python. The comparison is made by using two classifiers, i.e., gated recurrent unit long short-term memory (GRU-LSTM) classifier with one layer of GRU having 200 neurons and one layer of LSTM having 100 neurons and deep neural network, long short-term memory (DNNLSTM) classifier with one layer of DNN having 200 neurons and one layer of LSTM having 100 neurons. Furthermore, we have compared our hybrid model with existing literature, as shown in Table 6. The system’s overall performance improves by the quick multiplication of matrixes and is also carried out by Cu-DNNGRU + Cu-BLSTM. Table 2 depicts a thorough description of the proposed DL classifiers.

### 3.3. Data Set

The selection of an appropriate data set significantly contributes in evaluating the performance of a threat detection scheme. In the existing literature, the authors used various data sets, i.e., NSLKDD, KDD99, and few others, for threat detection in the IoT environment. However, most of these data sets lack the supportive features of IoT. Some attackers scan for local devices of IoT by creating web pages for taking control of these devices. Furthermore, they use malevolent scripts as well as DNS rebinding for discovering and attacking local IoT devices [46]. Therefore, the proposed work used state of art publicly available data set, CICIDS 2018 [47]. This data set consists of IoT supportive features, i.e., network flow features. Furthermore, it consists of benign as well as threat samples and is multiclass. It has seven categories and 14 up-to-date attacks (i.e., brute force, DDoS, botnet, bot, etc.) and more than 80 traffic features. However, in the proposed work, the total distribution is across six different classes, which include benign and attacks. Furthermore, we have selected all the features of this data set. The data set comprises 84,702 instances: 69,654 are benign, and the remaining 15,138 are instances of attacks. Detailed information on these classes of attacks and benign is given in Table 3.

### 3.4. Data Set Preprocessing

The data in the data set is presented in diverse forms, so it is not reliable to directly feed this data for classification to an algorithm. Firstly, we have deleted all the rows that had blank and nan values as it can influence the data quality and the evaluation model. DL algorithms mainly process the numeric data; thus, we have transformed all the nonnumeric values to numeric values through label encoder, i.e., sklearn. Furthermore, one-hot encoding has been performed on the output label as the category ordering can also reduce the model performance, leading to unexpected results. To increase the model effectiveness, data normalization is also conducted. We have used the MinMax scalar function on the data set.

## 4. Experimental Setup

We used an Intel processor, Core i7-7700, and graphics processing unit (GPU) for the purpose of experimentation. Furthermore, the proposed module is trained using Keras with the 3.8 version of Python. Table 4 depicts a complete specification of software and hardware.

### Standard Evaluation Metrics

The performance of the proposed architecture is evaluated using the standard metrics of evaluation, such as accuracy, F1-score, recall, precision, etc. However, for the calculation of specific parameters, first, we need to compute the false positive (FP), true positive (TP), false omission (FOR), Matthews correlation coefficient (MCC), false negative (FN), and true negative (TN).

(1) $$Accuracy=\frac{Tpos+Tneg}{Tpos+Tneg+Fpos+Fneg}$$

(2) $$Recall=\frac{Tpos}{Tpos+Fneg}$$

(3) $$Precision=\frac{Tpos}{Tpos+Fpos}$$

(4) $$F1-score=\frac{2*Tpos}{2*Tpos+Fpos+Fneg}$$

## 5. Results and Discussion

A complete outcome of the proposed hybrid model (Cu-DNNGRU + Cu-BLSTM) is presented in this section. For a thorough performance evaluation of our proposed hybrid model, we made the comparison of our model with our constructed two DL-driven hybrid models, i.e., Cu-GRULSTM and DNNLSTM, and with existing literature. The following standard evaluation metrics evaluate the proposed model.

### 5.1. Confusion Matrix Analysis

**Figure 3:**
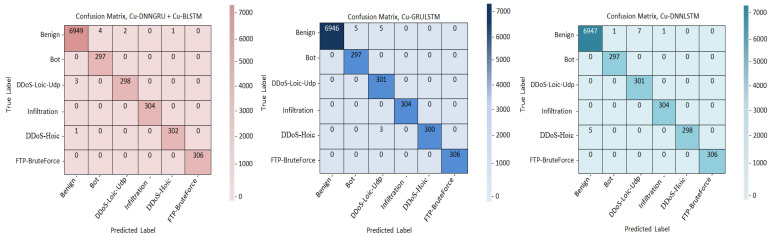
Confusion metrics of Cu-DNNGRU + Cu-BLSTM, Cu-GRULSTM, and Cu-DNNLSTM.

It is used for showing the classification model output. A complete analysis of the confusion matrix shows that Cu-DNNGRU + Cu-BLSTM identifies classes properly. The figure shows the confusion metrics of all of the three models. Figure 3 demonstrates that the proposed model Cu-DNNGRU + Cu-BLSTM recognizes the classes correctly and outperforms the other two architectures, i.e., Cu-GRULSTM and Cu-DNNLSTM.

### 5.2. Cross-Validation

To prove the unbiasedness of our outcomes, we have used the 10-fold cross-validation. Table 5 depicts a thorough description of each fold. However, for evaluation metrics, the average results of 10 folds are presented in several parts of this research work.

### 5.3. Roc Curve Analysis

**Figure 4:**
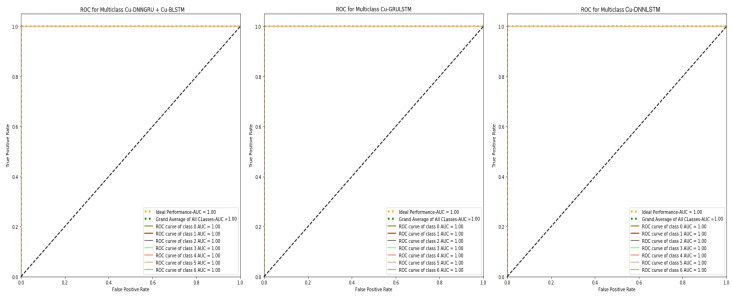
ROC Curves of Cu-DNNGRU + Cu-BLSTM, Cu-GRULSTM, Cu-DNNLSTM.

In any intrusion detection system (IDS), the Roc is considered an important parameter. The Roc plots the results for comparing the true negative rates (TNR) and true positive rates (TPR). The Roc curves of our proposed models is shown in Figure 4, which clearly show the relation of true positives and true negative.

### 5.4. Accuracy, Recall, F1-Score, and Precision

**Figure 5:**
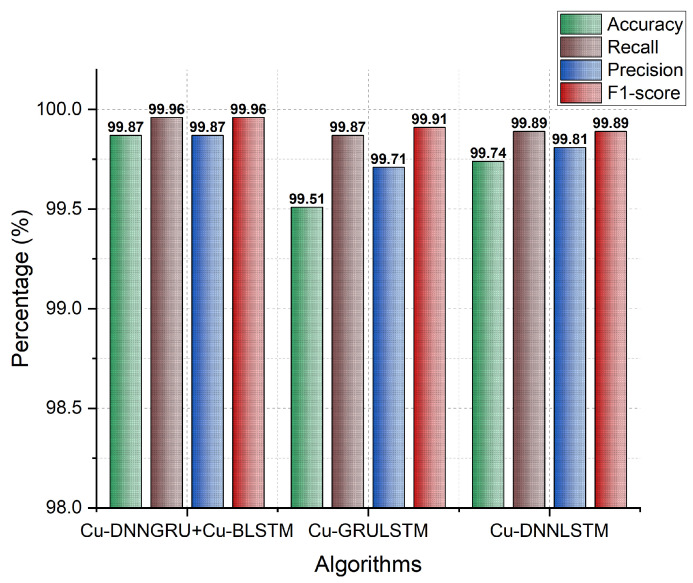
Accuracy, Recall, F1-score, and Precision.

Accuracy demonstrates the performance and efficiency of a classifier. It shows the amount of samples which is appropriately recognized by the model. The accuracy of our proposed model, i.e., Cu-DNNGRU + Cu-BLSTM, is shown in Figure 5. The hybrid model achieved an accuracy of 99.87% with a recall of 99.96%. The precision indicates the number of records that are identified correctly. The precision of our proposed model is 99.87%, with an F1-score of 99.96%. The detailed results for each fold are shown in Table 5 for accuracy, precision, F1-score, and recall.

### 5.5. FPR, FOR, FNR, and FDR Analysis

**Figure 6:**
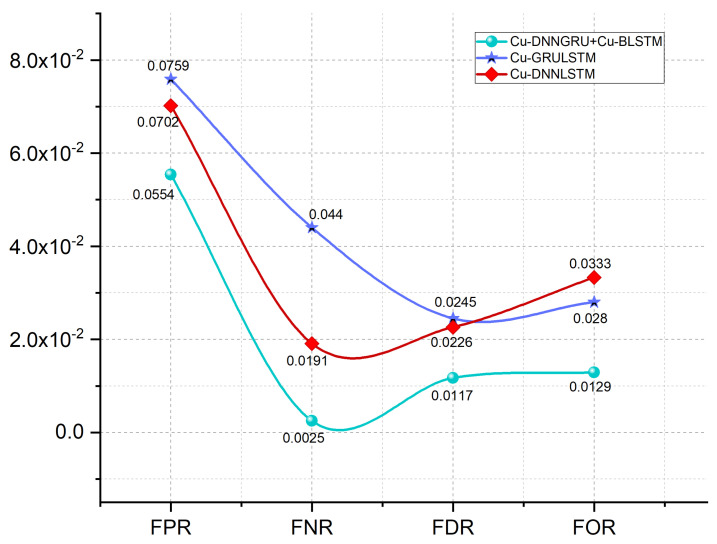
Achieved values of FPR, FNR, FDR, and FOR.

For an enhanced assessment of our proposed hybrid model, we have estimated the false positive rate (FPR), false omission rate (FOR), false discovery rate (FDR), and false negative rate (FNR). Figure 6 depicts the results of these metrics, which shows that our proposed model Cu-DNNGRU + Cu-BLSTM has FPR and FOR of 0.0554% and 0.0129% with 0.0025% and 0.0117% of FNR and FDR. Thus, the proposed model shows better results than the other two models, as shown in Figure 6. Furthermore, the results of DNNLSTM are better than GRULSTM.

### 5.6. TNR, TPR, and MCC Analysis

**Figure 7:**
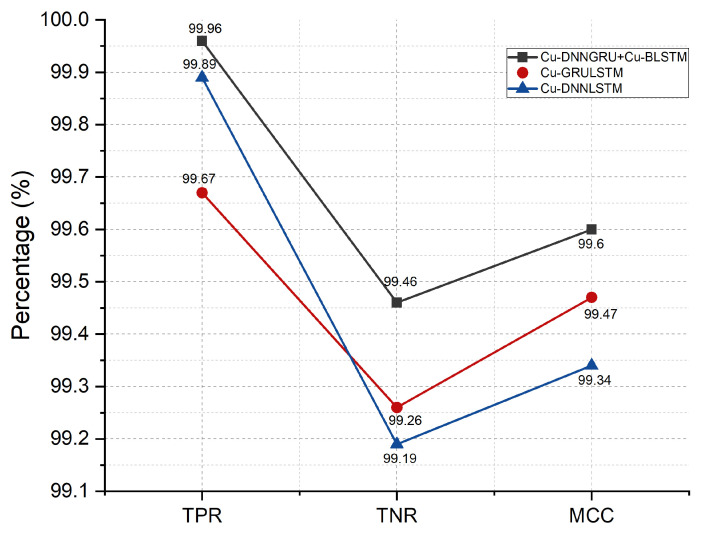
TNR, TPR, and MCC.

For a thorough analysis and evaluation of the proposed model, a confusion matrix is used for getting the values of TNR, TPR, and MCC, respectively. Figure 7 depicts the scores of Tpr, Tnr, and MCC, which are 99.96%, 99.43%, and 99.60%. The proposed model has better outcomes, as shown in Figure 7.

### 5.7. Speed Efficiency

**Figure 8:**
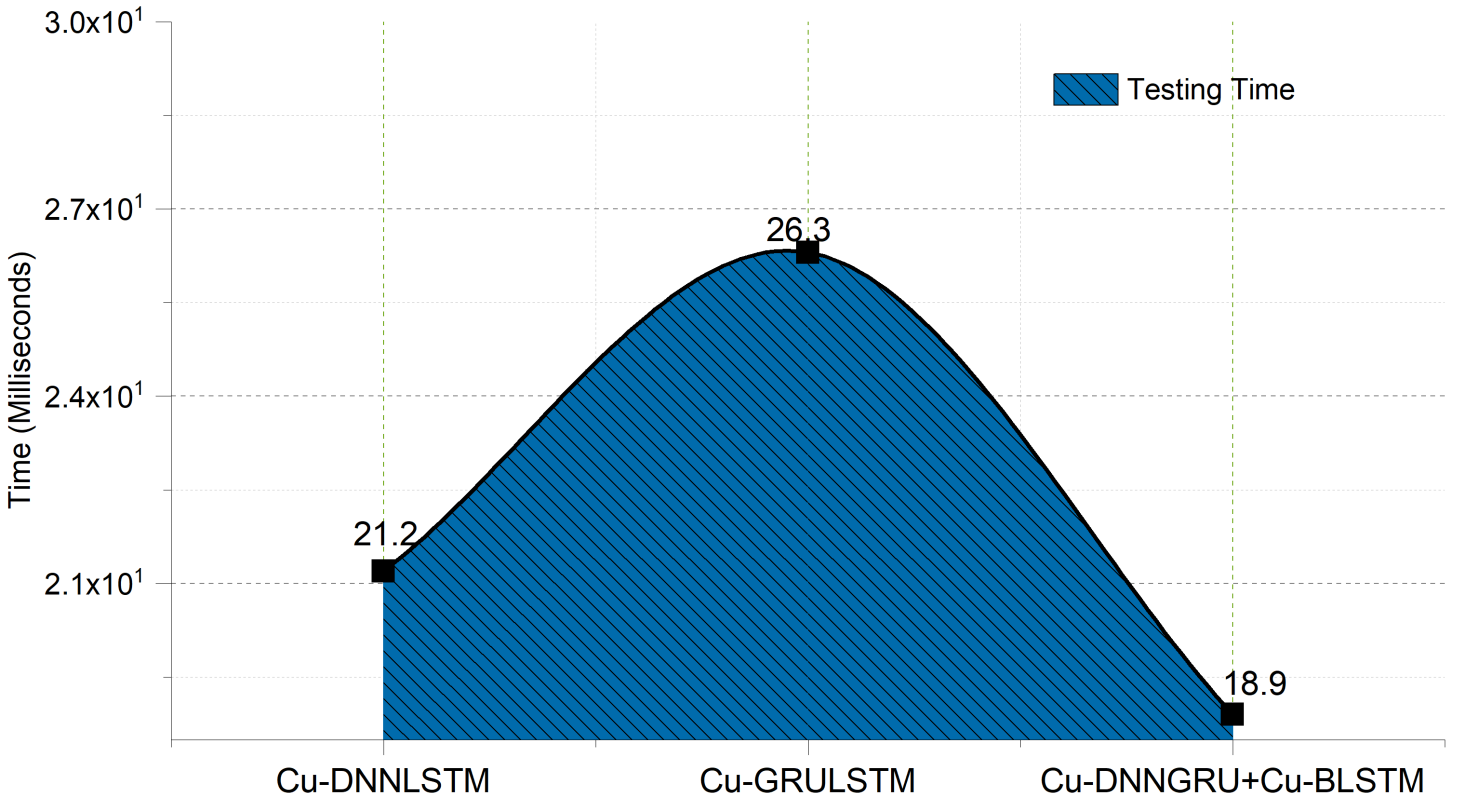
Testing time of CU-DNNGRU + Cu-BLSTM, Cu–DNNGRU, and Cu-DNNLSTM.

The testing time of the proposed model is shown in Figure 8. As the training phase is mainly done offline, so it is not considered. On the other hand, the testing phase is considered important as it demonstrates efficiency and the model’s performance. The proposed hybrid model has a good testing time of 18.90 ms, proving that our proposed model, i.e., Cu-DNNGRU and Cu-BLSTM is computationally efficient. Furthermore, the testing time of DNNLSTM is less than GRULSTM.

### 5.8. Proposed Model Comparison with Existing DL Algorithms

To show the efficiency of our proposed model, i.e., Cu-DNNGRU + Cu-BLSTM, we used two other hybrid DL models (i.e., Cu-GRULSTM and Cu-DNNLSTM) in this work for the purpose of comparison. Both of these models are trained on the CICIDS 2018 data set with the same metrics of evaluation. Table 2 shows the complete architecture of these models. Furthermore, we have also made the comparison of our proposed model with existing benchmark algorithms. The comparison with current benchmarks is presented in Table 6. The proposed model, i.e., Cu-DNNGRU + Cu-BLSTM, delivers better results in evaluation metrics, i.e., accuracy, F1-score, precision, etc., and speed efficiency. In addition, Cu-DNNGRU + Cu-BLSTM shows a testing time of only 18.9 (ms), which is comparatively less than the existing benchmarks.

## 6. Conclusions

IoT demands a flexible, reliable, and secure infrastructure. Recently, deep learning gained the attention of the world through its advancement. In this paper, an SDN-enabled, hybrid DL-driven architecture is proposed to protect the IoT environment against malware and cyberattacks, i.e., DDoS, bruteforce, bot, and infiltration. We have used state-of-the-art Cuda-DNNGRU and Cuda-BLSTM classifiers for effective threat detection. The proposed architecture is cost-effective as well as highly scalable. Furthermore, the results of our proposed model are compared with two other hybrid algorithms that are trained and evaluated on the same data set, i.e., Cuda-GRULSTM and Cuda-DNNLSTM. The results are evident, that the proposed model beats the results of these two hybrid models and current benchmarks.The performance advantages of the model are verified by comparing the evaluation metrics of accuracy, recall, precision, F1 Score and speed efficiency. The proposed model achieved 99.87% accuracy with FPR of 0.0554%, and testing time of only 18.9 ms which is relatively better than the existing literature, proving the efficiency of our proposed model in terms of speed efficiency and detection accuracy. In the future, the authors aim to utilize hybrid deep learning algorithms along with SDN and blockchain for intrusions and threats detection in IoTs. Finally, we conclude that the hybrid models of deep learning play an essential role in the security of the IoT environment.

## Figures and Tables

**Table 1:** Comparison of existing literature.

Ref	Algorithm	Approach	Data Set	D.Accuracy	Time Complexity
[14]	LSTM	Cyber threats detection in a smart device using a deep learning model	Modbus-TCP	High	High
[15]	RNN, LSTM, and GRU	Presented ML and DL techniques for intrusion detection	KDDCUP99	Low	N/A
[16]	RF	Presented a technique using ML classifier for DDoS attack detection in IoT	Self-generated data set by using Wireshark	High	N/A
[17]	SVM	Proposed an ML technique for IDS in SDN	DARPA	Medium	N/A
[18]	GRU	Proposed a self-learning distribution for identifying infected smart devices	Real Shelf Consumer IoT devices	Low	Medium
[19]	LSTM	Proposed a deep-learning-driven technique for botnet detection	CVUT real-time traffic	High	N/A
[20]	Bayesian, J48, naïve Bayes	Presented a machine learning approach for IRC botnet detection	Dartmouth wireless network	Low	N/A
[21]	LSTM-RNN	Propose an ML-driven approach to detected known and unknown threats	NSL-KDD	Low	N/A
[22]	ANN	Presented ANN learning procedures for intrusion detection by using feed-forward and back learning algorithms	Internet packet traces	High	N/A
[23]	Deep model	Presented a DL-driven scheme in IoT for the detection of DoS attacks.	NSL-KDD	Medium	Medium
[24]	RBM	SDN-based DL technique for DoS attacks detection in intelligent devices	KDD99	Low	N/A
[27]	RTS-DELM-CSIDS	Presented ML-based approach to develop an intrusion detection system	NSLKDD	Low	High
[28]	DNN, SVM, J48 and Naivebayes	Presented different algorithms to improve the learning rate of the algorithm, which can predict attacks in IDS	NSL-KDD	Low	N/A
[29]	CNN and RNN	The proposed methodology can detect botnets at the packet level	ISOT and CTU-13	Low	High

**Table 2:** Hybrid algorithms description.

Algorithm	Layers	AF	Neurons	LF	Optimizer	Batch-Size	Epochs
Cu-DNNGRU + Cu-BLSTM	Cu-DNNGRU (1)	Relu	(200)	CC-E			
	Cu-BLSTM (1)	Relu	(100)	CC-E			
	Dropout	–	(0.3)	–	Adamax	32	05
	Output Layer (1)	Softmax	07				
	Dense (3)	–	(200,100,50)	–			
Cu-GRULSTM	GRU Layer (1)	Relu	(200)	CC-E			
	LSTM Layer (1)	Relu	(100)	CC-E			
	Dropout	–	(0.3)	–	Adamax	32	05
	Dense (3)	–	(200,100,50)	–			
	Output Layer (1)	Softmax	07				
Cu-DNNLSTM	DNN Layer (1)	Relu	(200)	CC-E			
	LSTM Layer (1)	Relu	(100)	CC-E			
	Dropout	–	(0.3)	–	Adamax	32	05
	Dense (3)	–	(200,100,50)	–			
	Output Layer (1)	Softmax	07				

**Table 3:** Data Set Description, CICIDS2018.

Classes	Attack	Instances
Benign	–	69,654
Bot	–	2977
Brute Force	FTP	3066
DDoS	Loic-UDP	3015
	Hoic	3037
Infiltration	–	3043
Total		84,702

**Table 4:** Experimental setup.

CPU	7700, i7, 7th Generation with 2.80 GHz processor
OS	Windows 10, 64 Bit
GPU	Nvidia GeForce 1060 6 GB
RAM	16 GB
Libraries	Pandas, TensorFlow, Numpy, Scikitlearn, and Keras
Language	Python with version 3.8

**Table 5:** 10-folds cross validation results.

Parameter	DL Models	1	2	3	4	5	6	7	8	9	10
**Accuracy (%)**	*Cu-DNNGRU + Cu-BLSTM*	99.81	99.77	99.85	99.91	99.88	99.90	99.90	99.90	99.92	99.87
	Cu-GRULSTM	98.85	99.83	99.81	98.86	98.59	99.72	99.15	99.56	99.84	99.85
	Cu-DNNLSTM	99.81	99.85	99.81	99.74	99.72	99.71	99.72	99.74	99.62	99.71
**F1-score (%)**	*Cu-DNNGRU + Cu-BLSTM*	99.97	99.91	99.98	99.98	99.91	100	100	100	100	99.94
	Cu-GRULSTM	99.89	99.92	99.95	99.95	99.96	99.98	99.65	99.95	99.91	99.95
	Cu-DNNLSTM	99.92	99.89	99.95	99.89	99.97	99.91	99.94	99.88	99.81	99.82
**Recall (%)**	*Cu-DNNGRU + Cu-BLSTM*	99.97	99.91	99.98	99.98	99.91	100	100	100	100	99.94
	Cu-GRULSTM	99.89	99.92	99.95	99.95	99.45	99.86	99.95	99.89	99.91	99.95
	Cu-DNNLSTM	99.92	99.89	99.95	99.89	99.83	99.87	99.86	99.89	99.90	99.91
**Precision (%)**	*Cu-DNNGRU + Cu-BLSTM*	99.79	99.81	99.84	99.91	99.94	99.88	99.88	99.88	99.91	99.89
	Cu-GRULSTM	99.85	99.87	99.81	99.18	99.66	99.84	99.85	99.78	99.76	99.51
	Cu-DNNLSTM	99.84	99.85	99.85	99.88	99.69	99.76	99.69	99.88	99.82	99.87

**Table 6:** Comparison of proposed model with existing literature.

Ref	Data Set	Accuracy	T.Time	Algorithm	10 Fold	Cu-E	Precision	F1-Score	Recall
***Proposed model***	CICIDS2018	99.87%	18.9 ms	Cu-DNNGRU + Cu-BLSTM	√	√	99.87%	99.96%	99.96%
[48]	CICIDS2018	91.50%	–	CNN	–	–	–	–	–
[49]	CICIDS2017	89.00%	–	GRU-RNN	–	–	99.00%	99.00%	99.00%
[50]	CICIDS2017	98.60%	296 ms	LSTM-CNN	√	√	99.37%	99.35%	99.50%
[51]	CICIDS2018	96.11%	–	2L-ZED-IDS	–	–	93.20%	–	96.90%

## Data Availability

Not applicable.

## Abbreviations

The following abbreviations are used in this manuscript

IoT	Internet of things
SVM	Support vector machine
DNN	Deep neural network
GRU	Gated recurrent unit
ANN	Artificial neural network
LSTM	Long short term memory
SDN	Software-defined Networking
API	Application programming interface
DoS	Denial of service
DDoS	Distributed denial of service
BLSTM	Bidirectional long short term memory
IDS	Intrusion detection system
RF	Random forest
RNN	Recurrent neural network
TCP	Transfer control protocol
IRC	Internet relay chat
RBM	Restricted boltzmann machine
DL	Deep learning
DAE	Deep autoencoder
CNN	Convolutional neural network
LOIC	Low orbit ion cannon
DNS	Domain name system
UDP	User datagram protocol
HOIC	High orbit ion cannon
DFFNN	Deep feed forward neural network
ROC	Receiver operating characteristic
AF	Activation function
LF	Loss function
Relu	Rectified linear unit
