# Correction: Javeed et al. A Hybrid Deep Learning-Driven SDN Enabled Mechanism for Secure Communication in Internet of Things (IoT). *Sensors* 2021, *21*, 4884

**DOI:** 10.3390/s25185739

**Published:** 2025-09-15

**Authors:** Danish Javeed, Tianhan Gao, Muhammad Taimoor Khan, Ijaz Ahmad

**Affiliations:** 1 Software College, Northeastern University, Shenyang 110169, China; 2027016@stu.neu.edu.cn; 2 Riphah Institute of Science and Engineering, Islamabad 44000, Pakistan; mtaimoor.khan@infosecurity.com.pk; 3 Institute of Computer Sciences and Information Technology (ICS/IT), The University of Agriculture, Peshawar 25130, Pakistan; ijaz@siat.ac.cn

**Affiliation Correction**

In the published publication [1], there was an error regarding the affiliation “Shenzhen Institute of Advanced Technology, Chinese Academy of Sciences, Shenzhen 518000, China” for Ijaz Ahmad due to improper authorization. The updated affiliation should be “Institute of Computer Sciences and Information Technology (ICS/IT), The University of Agriculture, Peshawar 25130, Pakistan”.

The authors state that the scientific conclusions are unaffected. This correction was approved by the Academic Editor. The original publication has also been updated.
